# Determinants of Prolonged Antibiotic Administration in Culture-Negative Evaluations of Early-Onset Neonatal Meningitis: A Retrospective Cohort Study

**DOI:** 10.3390/antibiotics14090925

**Published:** 2025-09-12

**Authors:** Rowan Mesilhy, Ibrahim Safra, Shaikha Alnaimi, Ala Ali, Rayan Terkawi, Mohammed Gaffari, Talal Alhendawi, Anvar P. Vellamgot, Ashraf Gad

**Affiliations:** 1 Department of Internal Medicine, Hamad Medical Corporation, Doha P.O. Box 3050, Qatar; rawanmesilhy@gmail.com; 2 Neonatal Intensive Care Unit, Critical Care Department, Women’s Wellness and Research Center, Hamad Medical Corporation, Doha P.O. Box 3050, Qatar; isafra@hamad.qa (I.S.); avellamgot@hamad.qa (A.P.V.); 3 Pharmacy Department, Women’s Wellness and Research Center, Hamad Medical Corporation, Doha P.O. Box 3050, Qatar; salnaimi4@hamad.qa; 4 Department of Pediatrics, The View Hospital, Doha P.O. Box 201184, Qatar; al.ali@theviewhospital.com; 5 Department of Pediatrics, Ann & Robert H Lurie Children’s Hospital of Chicago, Chicago, IL 60611, USA; rterkawi@luriechildrens.org; 6 Department of Neonatology, Nottingham University Hospitals NHS Trust, Nottingham NG7 2UH, UK; mohammed.gaffari1@nhs.net; 7 Critical Care Medicine, Department of Pediatrics, American Family Children’s Hospital, University of Wisconsin—Madison, Madison, WI 53792, USA; talal_hindawi@hotmail.com

**Keywords:** neonatal meningitis, sepsis, prolonged antibiotic therapy, C-reactive protein, CRP, cerebrospinal fluid, CSF

## Abstract

**Background:** Early-onset neonatal meningitis (EONM) is a rare but serious condition where antibiotics are often given for extended periods, even without a positive cerebrospinal fluid (CSF) culture. The reasons for this prolonged treatment are unknown. **Methodology:** This study, conducted at the Women’s Wellness and Research Center, Doha, retrospectively analyzed the determinants of prolonged antibiotic therapy among neonates with sterile CSF cultures during the first week of life, born during 2015 to 2018. **Results:** Of 315 neonates without confirmed meningitis, 96 (30.5%) received prolonged antibiotic therapy. These infants had significantly lower birth weights (2790 g vs. 3170 g) and gestational ages (36.7 weeks vs. 38.5 weeks). They were more likely to require respiratory support, appear ill, and have laboratory abnormalities, including neutropenia, positive blood cultures (36.5% vs. 0.9%), elevated C-reactive protein (CRP), and higher CSF protein. Multivariable analysis identified low Apgar scores (Adjusted Odds Ratio (aOR), 2.82), positive blood cultures (aOR, 118.48), traumatic lumbar puncture (LP) (aOR, 2.14), CRP levels ≥ 50 mg/L (aOR, 2.60), delayed LP (OR, 8.28), and elevated cerebrospinal fluid white cell counts (aOR, 5.47) as independent predictors of prolonged antibiotic use. **Conclusions:** Prolonged antibiotic use in neonates with sterile CSF cultures and suspected EONS is common and may be driven by certain clinical and laboratory indicators of illness severity and inflammation. Identifying these predictors can support risk-stratified treatment decisions, promoting safer antimicrobial stewardship.

## 1. Introduction

Neonatal sepsis remains a significant cause of morbidity and mortality, especially in premature infants, yet achieving a timely and accurate diagnosis remains challenging due to nonspecific clinical signs and the absence of unified diagnostic criteria, particularly in preterm neonates [1]. Early-onset neonatal meningitis (EONM), defined as meningitis occurring within the first 72 h of life, is a debilitating form of neonatal sepsis with mortality and morbidity rates of 10% and 20–50%, respectively [2]. Although the incidence of culture-confirmed EONM in term infants is estimated at 0.02 to 0.04 per 1000 live births, the burden rises significantly among preterm neonates, reaching approximately 0.7 cases per 1000 live births at 22–28 weeks’ gestation [3].

The clinical features of meningitis in neonates—such as temperature instability, apnea, irritability, or feeding difficulties—frequently overlap with those of sepsis, rendering differentiation difficult. Consequently, lumbar puncture (LP) for Cerebrospinal Fluid (CSF) analysis remains a key diagnostic procedure, despite ongoing debate regarding its timing and necessity. The American Academy of Pediatrics (AAP) recommends LP in critically ill neonates or those with a positive blood culture, while also recognizing that most term neonates with sterile blood cultures may not require it [4]. While the vast majority of term infants do not need LP if blood cultures are sterile, 1–2 cases per 100,000 live births will have culture-confirmed meningitis. Therefore, the decision to perform LP in the absence of documented bacteremia in such cases, especially in preterm infants, depends on the treating physician’s clinical judgment [3,4]. Biomarkers such as C-reactive protein levels (CRP) have been proposed as adjunctive indicators to guide LP decisions. For instance, the UK’s National Institute for Health and Care Excellence (NICE) recommends performing an LP if CRP exceeds 10 mg/L [5]. However, this cut-off has led to an unnecessary 30-fold increase in LPs in 59% of trusts that adopted the NICE guideline, despite missing 11% of meningitis cases [6]. In this study, the sensitivity of CRP > 10 mg/L, performed 4 days around the positive LP, was 89%. This value is likely to be lower if CRP testing was limited to <24 h, as per NICE CG149 guidelines [5]. In other studies, CRP > 40 mg/L demonstrated poor sensitivity and specificity but a relatively high negative predictive value (85–90%), while a higher cut-off of >80 mg/L yielded greater specificity (85%) with moderate sensitivity (67%) for guiding LP decisions [7,8]. Therefore, performing LPs based solely on serum CRP values of different cut-offs remains questionable, especially when evaluating EOS compared to late-onset sepsis [9]. The finding that up to 38% of CSF culture-proven meningitis cases have negative blood culture further complicates this diagnostic dilemma of EONM [10]. Additionally, no single CSF value is reliable in excluding the presence of neonatal meningitis.

At our center, among infants with suspected early-onset sepsis, a LP was performed when there were clinical signs of meningitis, a positive blood culture, or increasing CRP. For the purposes of this study, infants were classified as having culture-negative EONM if CSF abnormalities were present without a positive CSF culture or Polymerase Chain Reaction (PCR).

In many settings, CSF cultures often return negative results, either due to prior antibiotic exposure, delayed sampling, or a low pathogen burden. In such culture-negative evaluations, clinicians are frequently compelled to continue empiric antibiotic therapy beyond the standard 48–72 h, often extending to 7–14 days, in fear of missing occult or partially treated meningitis [11]. While this cautious approach is understandable, prolonged antibiotic administration in neonates is not without risk. Excessive exposure has been linked to dysbiosis, an increased risk of necrotizing enterocolitis in preterm infants, the selection of multidrug-resistant organisms, and more extended hospital stays [12,13]. Furthermore, antimicrobial stewardship efforts emphasize the importance of minimizing unnecessary antibiotic use, especially in vulnerable populations such as neonates. Despite this, limited evidence exists on the specific determinants that drive prolonged antibiotic use in neonates with culture-negative CSF evaluations during early-onset sepsis workups.

This gap underscores the need to clarify how clinical, laboratory, and institutional factors shape antibiotic decision-making in these situations. Identifying these determinants can inform more targeted stewardship strategies, optimize antibiotic use, and reduce iatrogenic harm. Therefore, this study aims to identify the clinical, laboratory, and CSF factors associated with prolonged antibiotic therapy (beyond seven days) in neonates undergoing evaluation for meningitis in the first week of life with negative CSF cultures.

## 2. Results

**Figure 1:**
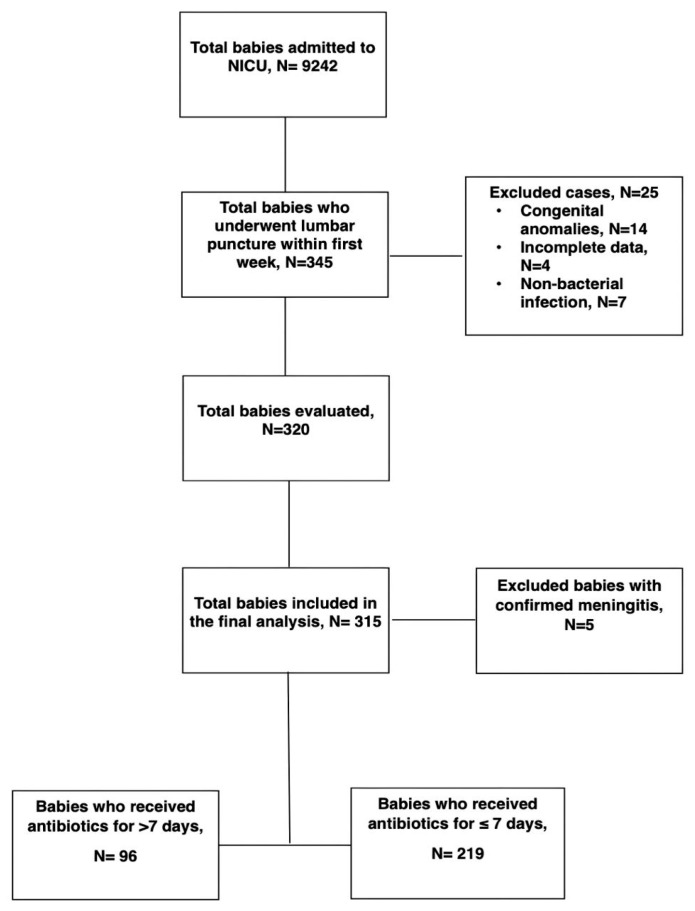
Flow chart of the study population.

We identified a total of 9242 babies who were admitted to the NICU during the study period. Of these, 345 neonates underwent LP within the first seven days of life. Twenty-five infants were excluded based on predefined exclusion criteria, including congenital anomalies (n = 14), incomplete or missing data (n = 4), or confirmed non-bacterial infections (four cases of congenital syphilis, two toxoplasmosis, and one herpes simplex). A total of 320 neonates met the eligibility criteria for complete evaluation. Among them, five neonates were diagnosed with culture-confirmed meningitis and were excluded from the final analysis. The remaining 315 infants with negative CSF cultures were included in the study cohort. Among them, 96 infants (30.5%) received prolonged antibiotics (>7 days), while 219 (69.5%) received a shorter course (≤7 days) (Figure 1).

The baseline characteristics of both groups are summarized in Table 1. Neonates who received prolonged antibiotics (n = 96) had a significantly lower mean birth weight (BW) compared to those who received a shorter course (n = 219) (2797.5 ± 857.2 g vs. 3168.4 ± 606.7 g, *p* < 0.001). Similarly, the median gestational age (GA) was lower in the prolonged group (38.0 weeks [interquartile range (IQR): 35–40]) vs. 39.0 weeks [IQR: 38–40] in the short-course group (*p* < 0.001). Preterm birth was more prevalent among infants receiving prolonged therapy (32.3% vs. 14.2%, Odds Ratio (OR) = 2.89, 95% CI: 1.63–5.13, *p* < 0.001). A low Apgar score at 1 min (<7) was significantly more common in the prolonged group (31.6% vs. 12.8%; OR = 3.15, 95% CI: 1.75–5.66; *p* < 0.001), along with increased rates of delivery room intubation (9.4% vs. 1.8%; OR = 5.56, 95% CI: 1.67–18.53; *p* = 0.004) and respiratory support in the delivery room (40.6% vs. 22.8%; OR = 2.31, 95% CI: 1.38–3.87; *p* = 0.001). Furthermore, these infants were more likely to require respiratory support beyond the delivery room (62.5% vs. 40.6%; OR = 2.43, 95% CI: 1.49–3.99; *p* < 0.001) and oxygen therapy (62.5% vs. 41.1%; OR = 2.39, 95% CI: 1.46–3.91; *p* < 0.001), with a significantly longer median duration of respiratory support (44.5 vs. 0 h; *p* < 0.001). Hypoactivity (12.5% vs. 5.0%; OR = 2.70, 95% CI: 1.15–6.36; *p* = 0.019), ill appearance at presentation (62.5% vs. 47.0%; OR = 1.88, 95% CI: 1.15–3.07; *p* = 0.011), and presence of two or more systemic symptoms (64.5% vs. 50.0%; OR = 1.82, 95% CI: 1.09–3.02; *p* = 0.021) were also significantly more prevalent among neonates who received prolonged therapy. Traumatic LP (≥500 red blood cells (RBCs)) was more frequent in the prolonged group (45.9% vs. 31.5%; OR = 1.84, 95% CI: 1.09–3.13; *p* = 0.023). Female sex, ethnicity, intrauterine growth restriction, and abnormal brain neuroimaging findings (ultrasound or MRI) did not significantly differ between groups. Mortality was rare in both groups, with only two deaths in the prolonged group (2.1%), and the difference was not statistically significant (*p* = 0.092).

Maternal demographic and clinical characteristics were generally comparable between the two groups, as detailed in Table 2. No significant differences were observed in gravidity, parity, group B Streptococcus (GBS) status, or chorioamnionitis. The use of intrapartum antibiotics ≥4 h before delivery (48.6% vs. 40.6%, *p* = 0.19) and the presence of prolonged rupture of membranes (>24 h) were similar across groups. Likewise, maternal temperature and CRP values within 24 h before or after delivery did not differ significantly. Although not statistically significant, a higher proportion of positive placental cultures was observed in the prolonged antibiotic group (81.5% vs. 63.9%, OR = 2.49, *p* = 0.093). Similarly, positive maternal blood cultures were more frequent in this group (17.5% vs. 8.4%, OR = 2.31, *p* = 0.139). The mode of delivery was not different between the groups.

Significant differences were observed in laboratory and CSF parameters between neonates who received prolonged antibiotic therapy and those who received ≤7 days, as shown in Table 3. Positive blood cultures were markedly more common in the prolonged group (36.5% vs. 0.9%, OR = 62.25, 95% CI: 14.44–266.19, *p* < 0.001), as were positive cultures obtained within the first 28 days (27.1% vs. 0.9%, OR = 40.3, *p* < 0.001). Inflammatory markers were also elevated: the highest CRP levels were ≥50 mg/L (47.9% vs. 31.5%, OR = 2.00, *p* = 0.005) and ≥100 mg/L (16.7% vs. 5.0%, OR = 3.78, *p* = 0.001), both of which were significantly associated with prolonged treatment. Low absolute neutrophil count was more frequent in the prolonged group (14.6% vs. 3.7%, OR = 4.50, *p* < 0.001). CSF findings revealed that infants in the prolonged group were more likely to have elevated CSF white cell counts (16.5% vs. 2.8%, OR = 6.94, *p* < 0.001), high CSF protein levels (33.7% vs. 18.0%, OR = 2.32, *p* = 0.005), and required repeat LP (14.6% vs. 1.8%, OR = 9.18, *p* < 0.001). LP timing was significantly later among infants receiving prolonged antibiotics (median 1.67 vs. 1.29 days, *p* = 0.004). There were no significant differences in CSF glucose levels, CSF-to-blood glucose ratios, or corrected (white blood cell (WBC) counts.

Multivariable logistic regression analysis identified several independent predictors of prolonged antibiotic therapy among neonates evaluated for early-onset sepsis, as outlined in Table 4. Notably, a positive blood culture was the strongest predictor, associated with a 118-fold increase in the odds of receiving antibiotics beyond 7 days (aOR: 118.5; 95% CI: 14.5–966.4; *p* < 0.001). Additionally, delayed LP timing beyond 72 h significantly increased the odds of prolonged treatment by over eightfold (aOR: 8.28; 95% CI: 3.24–21.16; *p* < 0.001) A traumatic LP (≥500 RBCs) was also associated with a more than two-fold increase in the odds of extended antibiotic use (aOR: 2.14; *p* = 0.045), likely due to diagnostic uncertainty. Similarly, elevated CSF white cell counts and CRP levels ≥50 mg/L were each associated with significantly increased odds—by approximately 5.5-fold and 2.6-fold, respectively—indicating that inflammatory markers have a substantial influence on treatment duration. Lastly, neonates with low GA and a 1 min Apgar score of ≤7 were associated with increased odds of receiving prolonged antibiotics (aOR: 0.9, *p* = 0.081; aOR: 2.82, *p* = 0.018, respectively).

## 3. Discussion

This retrospective cohort study investigated the factors associated with prolonged antibiotic administration (>7 days) in neonates undergoing evaluation for suspected early-onset meningitis with negative CSF cultures. Our findings reveal a multifactorial decision-making process, influenced by clinical, laboratory, and procedural parameters. The logistic regression model identified positive blood culture, traumatic LP (≥500 RBCs), elevated CSF white blood cell count, low GA, and low Apgar scores less than or equal to 7 at 1 min, high CRP levels, and delayed LP timing beyond 72 h as independent predictors of extended antibiotic therapy.

Notably, a positive blood culture was the most significant factor, increasing the odds of prolonged antibiotic use by over 118-fold. This finding highlights how clinicians often interpret bacteremia—even in the absence of a positive CSF culture—as a surrogate for invasive infection, warranting prolonged antibiotic coverage. Previous studies have similarly shown that blood culture positivity strongly influences antibiotic duration, reflecting a cautious approach in neonatal practice due to high morbidity associated with missed or partially treated sepsis and meningitis [11,14]. The timing of LP emerged as another critical determinant, with delays beyond 72 h increasing the odds of extended treatment by more than eightfold. This aligns with earlier findings where diagnostic uncertainty due to delayed CSF collection led to longer empirical treatment courses [15]. Clinical concern about partially treated meningitis often results in continuation of therapy despite sterile cultures, especially when antibiotics are started before CSF collection, potentially sterilizing the sample [16]. Traumatic LPs, with ≥500 RBCs, also predicted prolonged treatment. Blood-contaminated CSF samples are difficult to interpret and can mask true pleocytosis, complicating clinical decisions. This reflects a broader trend in NICU practice to err on the side of caution when CSF parameters are ambiguous [17]. Systemic and CSF inflammation, as indicated by elevated CRP levels (≥50 mg/L) and elevated CSF WBC counts, were also strongly associated with longer antibiotic durations. CRP is frequently used as a supportive marker in the diagnosis of neonatal sepsis and is often relied upon in ambiguous cases, despite its lack of specificity [18,19]. Elevated CSF WBC, a key indicator of CNS inflammation, naturally biases clinicians toward assuming meningitis, especially if uncorrected for traumatic taps, and can contribute to extended treatment durations [20]. Interestingly, low Apgar scores (<7 at 1 min) were independently associated with a nearly threefold increase in prolonged antibiotic use in cases of suspected EONM. This may reflect the perception that neonates, particularly very low BW neonates, with poor perinatal adaptation are at higher infection risk and thus receive more aggressive empiric therapy [21].

Our findings concur with previous reports showing variability and overuse in neonatal antibiotic prescribing, as neonates with suspected meningitis often receive prolonged therapy despite negative cultures, largely due to abnormal CSF findings or persistent systemic signs of illness [22]. Interestingly, maternal and perinatal factors including chorioamnionitis, GBS status, and maternal fever did not significantly predict prolonged antibiotic use, suggesting that postnatal clinical and laboratory findings play a more pivotal role in influencing treatment decisions. This diverges from earlier studies where maternal risk factors heavily dictated initial management but support a trend toward individualized postnatal risk stratification [16,23].

Our findings have several important implications for clinical practice. First, they highlight the persistent reliance on surrogate inflammatory markers and incomplete diagnostic data in guiding therapy, potentially leading to overtreatment. This highlights the importance of establishing clear institutional protocols that incorporate validated risk stratification tools to minimize unnecessary antibiotic exposure. Second, early and atraumatic LP remains essential for diagnostic clarity and antimicrobial stewardship. Third, as many of the identified predictors are modifiable (e.g., LP timing), quality improvement initiatives targeting procedural timing and technique may help reduce unnecessary antibiotic days.

### Strengths and Limitations

The study’s strengths are notable, as it stands as the first of its type to be executed in Qatar, providing crucial insights into antibiotic stewardship in culture-negative neonatal meningitis, a clinically ambiguous yet high-risk group. Furthermore, the use of regression analysis allows us to explore the determinants of prolonged antibiotic use, thereby enriching the study’s contributions to understanding the topic. These methodological considerations substantially augment the study’s integrity and the relevance of its findings within the field.

Despite its strengths, this study had limitations, including its retrospective design, which inherently restricts the ability to control for all potential confounding variables and limits causal inferences. Despite adjusting for key variables, unmeasured confounders—such as clinician preference, antibiotic choice, and evolving hospital protocols—may have influenced outcomes. Furthermore, our data were collected from a single center, which may limit their generalizability to other settings with different practices or patient populations. Lastly, while we focused on culture-negative meningitis, CSF viral PCR and other molecular diagnostics were not uniformly available, which may have influenced the duration of antibiotics and led to the missed detection of non-bacterial etiologies.

Future studies could address these limitations by incorporating a multicenter design to increase generalizability and include a prospective cohort to allow for a more controlled examination of the factors associated with prolonged antibiotic use. In addition, establishing validated clinical decision tools that integrate maternal risk factors, neonatal clinical presentation, and early biomarkers is crucial for better stratifying the risk of meningitis and guiding antibiotic duration in culture-negative cases. Furthermore, studies incorporating novel diagnostics—such as molecular assays and host-response biomarkers—may enhance diagnostic accuracy and reduce unnecessary interventions. Longitudinal follow-up is also warranted to evaluate neurodevelopmental outcomes and antimicrobial resistance patterns associated with different treatment durations.

## 4. Materials and Methods

### 4.1. Study Population and Sampling Strategy

This retrospective cohort study was conducted at the Neonatal Intensive Care Unit (NICU) of the Women’s Wellness and Research Center (WWRC) at Hamad Medical Corporation in Doha, Qatar. The study included neonates undergoing LP for evaluation of EONM within the first week of life and admitted to the NICU at the WWRC Hospital from January 2015 until December 2018.

Babies with significant congenital anomalies, culture-confirmed meningitis, and those with incomplete data were excluded from the study.

The cohort was subsequently categorized based on antibiotic duration: infants receiving antibiotics for more than 7 days were classified as part of the prolonged antibiotic group, while those treated for 7 days or fewer were considered part of the short-course group. The primary outcome of interest was the administration of prolonged antibiotic therapy, defined as treatment extending beyond 7 days in neonates with negative CSF cultures.

### 4.2. Study Variables

Clinical and demographic data were extracted from the electronic medical records. Neonatal variables included BW, GA, sex, Apgar scores at 1 and 5 min, delivery room interventions (e.g., intubation, respiratory support), body temperature, and presenting symptoms. Laboratory and CSF parameters included CRP levels, blood cultures, complete blood counts, corrected CSF WBC count, CSF protein, and glucose concentrations, as well as LP characteristics, such as timing and traumatic tap (defined as ≥500 RBCs per field).

Neutropenia was defined as an absolute neutrophil count < 1500 × 10^9^/L. CSF WBC > 26 × 10^9^/L in preterm and >23 × 10^9^/L in term, were presumed as abnormal [24,25,26]. Similarly, protein levels greater than 1.1 g/dL in term and greater than 1.5 g/dL in preterm were considered abnormal [27,28].

In traumatic samples, white blood cell counts were corrected by comparing them to the peripheral blood WBC: RBC ratio [29]. Similarly, 1.1 mg/dL was subtracted for every 1000 RBCs [30].

Maternal variables included parity, gravidity, mode of delivery, GBS status, chorioamnionitis, intrapartum antibiotic use, duration of rupture of membranes, and the highest maternal temperature or CRP before and after delivery. The primary outcome was prolonged antibiotic administration, defined as more than 7 days of antibiotic treatment in neonates with culture-negative cerebrospinal fluid results.

### 4.3. Statistical Analysis

Descriptive analyses were conducted to evaluate patient characteristics and clinical variables. Categorical variables were presented as frequencies and percentages and compared using the chi-square or Fisher’s exact test. Continuous variables were expressed as mean ± standard deviation or median with IQR and compared using the Student’s *t*-test or Mann–Whitney U test, as appropriate. A multivariable logistic regression model was employed to identify independent predictors associated with prolonged antibiotic use. We employed a stepwise approach that included all identified significant maternal and neonatal characteristics of interest, as well as short-term outcomes, including the primary outcome (*p*-value < 0.1), without data interaction. All *p*-values were two-tailed, and values below 0.05 were considered statistically significant. Data analysis was conducted using SPSS software, version 29.0 (IBM Corp., Armonk, NY, USA).

## 5. Conclusions

In this study of neonates with sterile CSF cultures during the first week of life, prolonged antibiotic therapy was prescribed in nearly one-third of cases. Infants who received extended treatment were more likely to appear clinically unstable, require respiratory support, and demonstrate abnormal laboratory findings. Independent predictors of prolonged antibiotic use included low Apgar scores, positive blood cultures, delayed or traumatic LP, elevated CRP, and increased CSF white cell counts.

The results suggest that clinicians determine the duration of antibiotic therapy using both clinical presentation and laboratory data, even in the absence of confirmed meningitis. While this strategy seeks to avoid missing cases of EONM, it increases the risk of unnecessary antibiotic exposure. Development of standardized, evidence-based protocols is required to optimize antibiotic management in neonates with suspected EONM.

## Figures and Tables

**Table 1:** Baseline Demographics and Clinical Characteristics of Neonates with Suspected Early-Onset Meningitis.

Variables	≤7 Days Antibiotics (n = 219)	>7 Days Antibiotics (n = 96)	Unadjusted OR *	95th CI	*p* Value
Birth weight, grams	3168.4 ± 606.7	2797.48 ± 857.2	370.930 *	179.89–561.96	<0.001
Gestational age, weeks	39.0 [38.0, 40.0]	38.0 [35.0, 40.0]			<0.001
Dry tap, n (%)	38 (17.4%)	11 (11.5%)	0.616	0.300–1.265	0.184
Traumatic tap, n (%)	57 (31.5%)	39 (45.9%)	1.844	1.086–3.132	0.023
Female sex, n (%)	102 (46.6%)	49 (51.0%)	1.196	0.740–1.933	0.465
Ethnicity, n (%)					0.424
Arab	129 (59.2%)	65 (67.7%)			
Caucasian	5 (2.3%)	2 (2.1%)			
Asian	70 (32.1%)	22 (22.9%)			
African	14 (6.4%)	7 (7.3%)			
Apgar at 1 min < 7 n (%)	28 (12.8%)	30 (31.6%)	3.148	1.751–5.662	<0.001
Apgar at 5 min < 7, n (%)	3 (1.4%)	5 (5.2%)	3.956	0.926–16.902	0.046
Delivery room intubation, n (%)	4 (1.8%)	9(9.4%)	5.560	1.668–18.532	0.004
Delivery room respiratory support, n (%)	50 (22.8%)	39 (40.6%)	2.313	1.381–3.871	0.001
Temp < 36 °C, n (%)	1 (0.5%)	2 (2.1%)	4.638	0.416–51.777	0.221
Temperature ≥ 38 °C, n (%)	11 (5.0%)	4 (4.2%)	0.822	0.255–2.650	1.000
Temperature change, n (%)	12 (5.5%)	6 (6.3%)	1.150	0.419–3.160	0.786
Hypoactivity, n (%)	11 (5.0%)	12 (12.5%)	2.701	1.147–6.361	0.019
Preterm birth, n (%)	31 (14.2%)	31 (32.3%)	2.892	1.632–5.125	<0.001
Low birth weight, n (%)	24 (11%)	26 (27.1%)	3.018	1.626–5.601	<0.001
IUGR,10th, n (%)	21 (9.6%)	10 (10.4%)	1.096	0.495–2.426	0.82
Resp support beyond delivery room, n (%)	89 (40.6%)	60 (62.5%)	2.434	1.486–3.987	<0.001
Duration of respiratory support, hours	0.0 [0.0, 24.0]	46.5 [0.0, 109.0]			<0.001
Oxygen beyond delivery room, n (%)	90 (41.1%)	60 (62.5%)	2.389	1.459–3.912	<0.001
Vasopressor use, n (%)	0 (0%)	2 (2.1%)	0.300	0.254–0.356	0.092
Suspected seizure/abnormal activity, n (%)	23 (10.5%)	6 (6.3%)	0.568	0.224–1.443	0.230
Well-appearing, n (%)	63 (28.8%)	20 (20.8%)	0.652	0.367–1.156	0.141
Ill-appearing, n (%)	103 (47.0%)	60 (62.5%)	1.877	1.149–3.067	0.011
Abnormal brain US, n (%)	10 (18.9%)	18 (32.1%)	2.037	0.838–4.949	0.113
Brain MRI, n (%)	15 (60.0%)	14 (82.4%)	3.111	0.707–13.689	0.124
Abnormal head US or MRI, n (%)	21 (38.2%)	24 (42.9%)	1.214	0.568–2.594	0.616
Death, n (%)	0 (0%)	2 (2.1%)	0.300	0.254–0.356	0.092

IUGR, intrauterine growth restriction; OR, odds ratio; CI, confidence interval. MRI—magnetic resonance imaging, US—ultrasound. * Mean difference.

**Table 2:** Maternal Baseline Demographics and Clinical Characteristics of Neonates with Suspected Early-Onset Meningitis.

Variables	≤7 Days Antibiotics (n = 219)	>7 Days Antibiotics (n = 96)	Unadjusted OR	95th CI	*p* Value
Gravidity, n (%)	103 (47.0%)	41 (42.7%)	0.840	0.518–1.362	0.478
Parity, n (%)	105 (47.9%)	46 (47.9%)	0.999	0.618–1.615	0.996
GBS status (positive or unknown), n (%)	141 (64.4%)	56 (58.3%)	0.774	0.474–1.266	0.307
Positive urine culture, n (%)	33 (17.6%)	13 (14.9%)	0.825	0.410–1.660	0.59
ROM > 24 h, n (%)	39 (17.8%)	16 (16.7%)	0.923	0.487–1.748	0.806
Meconium-stained liquor, n (%)	44 (20.2%)	23 (24.0%)	1.246	0.702–2.211	0.452
Chorioamnionitis, n (%)	95 (43.8%)	36 (37.5%)	0.771	0.471–1.261	0.299
Antibiotics ≥ 4 h before delivery, n (%)	106 (48.6%)	39 (40.6%)	0.723	0.445–1.176	0.19
Highest Temp 24 h before delivery, °C	37.508 ± 0.800	37.479 ± 0.827	0.0291 *	−0.1658–0.2240	0.769
Highest Temp 24 h after delivery, °C	37.164 ± 0.552	37.143 ± 0.664	0.0209 *	−0.1211–0.1628	0.772
Highest CRP 24 h before delivery, mg/L	33.50 [15.17, 63.50]	24.0 [11.00, 68.00]			0.280
Highest CRP 24 h after delivery, mg/L	73.0 [28.0, 161.0]	156.0 [29.2, 202.0]			0.454
Any antibiotics given, n (%)	127 (58.0%)	48 (50.0%)	0.724	0.447–1.173	0.189
Mode of delivery					0.143
Spontaneous Vaginal	82 (45.6%)	41 (48.2%)			
Elective CS	18 (10.0%)	9 (10.6%)			
Emergency CS	11 (6.1%)	0 (0.0%)			
Instrumental	69 (38.3%)	35 (41.2%)			
Positive placental culture, n (%)	46 (63.9%)	22 (81.5%)	2.487	0.842–7.350	0.093
Positive blood culture, n (%)	9 (8.4%)	7 (17.5%)	2.310	0.797–6.691	0.139
Blood culture organism					0.178
Negative results	98 (91.6%)	33 (82.5%)			
GBS	7 (6.5%)	5 (12.5%)			
*Escherichia coli*	0 (0.0%)	1 (2.5%)			
*Klebsiella pneumoniae*	1 (0.9%)	0 (0.0%)			
*Prevotella bivia*	0 (0.0%)	1 (2.5%)			
*Streptococcus pyogenes*	1 (0.9%)	0 (0.0%)			

OR, odds ratio; CI, confidence interval; GBS, Group B Streptococcus; ROM, rupture of membranes; CRP, C-reactive protein; CS, Cesarean section. * Mean difference.

**Table 3:** CSF and Laboratory Parameters of Neonates with Suspected Early-Onset Meningitis.

Variables	≤7 Days Antibiotics (n = 219)	>7 Days Antibiotics (n = 96)	Unadjusted OR	95th CI	*p*-Value
Blood Laboratory					
Low absolute neutrophil count (%)	8 (3.7%)	14 (14.6%)	4.503	1.821–11.135	<0.001
Positive blood culture	2 (0.9%)	35 (36.5%)	62.254	14.441–266.189	<0.001
First CRP ≥ 100, n (%)	8 (3.7%)	13 (13.5%)	4.131	1.652–10.331	0.001
Highest CRP ≥ 100, n (%)	11 (5.0%)	16 (16.7%)	3.782	1.683–8.500	0.001
First CRP ≥ 50, n (%)	64 (29.2%)	33 (34.4%)	1.269	0.760–2.117	0.362
Highest CRP ≥ 50, n (%)	69 (31.5%)	46 (47.9%)	2.0	1.223–3.270	0.005
CSF Parameters					
Lumbar puncture timing, days	1.60 ±1.0	2.13 ± 1.46	−0.536 *	−0.820, −0.251	<0.001
Elevated corrected CSF white cell count, n (%)	5 (2.8%)	14 (16.5%)	6.941	2.410–19.987	<0.001
High CSF protein concentrations, n (%)	32 (18.0%)	28 (33.7%)	2.323	1.282–4.209	0.005
Low CSF sugar or RBS, n (%)	11 (8.3%)	5 (7.8%)	0.94	0.312–2.829	0.912
Repeat CSF performed, n (%)	4 (1.8%)	14 (14.6%)	9.177	2.935–28.692	<0.001
High CSF WBC, n (%)	5 (2.8%)	14 (16.5%)	6.941	2.410–19.987	<0.001
Highest CRP, mg/L	37.0 [16.0, 63.0]	50.0 [25.25, 80.25]			0.002
Corrected CSF WBC, ×10^9^/L	2.99 [1.96, 7.99]	4.99 [1.99, 30.47]			0.006
CSF protein, mg/dL	77.5 [61.5, 100.0]	108.0 [74.0, 161.0]			0.008
CSF sugar, mmol/L	2.700 [2.40, 3.30]	2.900 [2.30, 3.50]			0.303
CSF sugar to blood sugar ratio	0.676 [0.49, 0.79]	0.736 [0.56, 0.89]			0.132

OR, odds ratio; CI, confidence interval; CRP, C-reactive protein; CSF, cerebrospinal fluid; WBC, white blood count. * Mean difference.

**Table 4:** Multivariable Logistic Regression Analysis of Factors Associated with Prolonged Antibiotic Use.

Variables	Adjusted OR	95th CI	*p*-Value
Traumatic lumbar puncture *	2.139	(1.016, 4.504)	0.045
Gestational age (weeks)	0.900	(0.8, 1.013)	0.081
Hypoactivity	3.436	(0.86, 13.726)	0.081
Late lumbar puncture (>72 h)	8.283	(3.243, 21.157)	<0.001
Low Apgar score at 1 min	2.818	(1.191, 6.667)	0.018
Positive blood culture	118.483	(14.527, 966.38)	<0.001
Corrected CSF WBC count	5.468	(1.336, 22.373)	0.018
Peak C-reactive protein ≥ 50 mg/L	2.596	(1.231, 5.474)	0.012

* CSF RBCs ≥ 500/mm^3^. RBC, red blood cell; CSF, cerebrospinal fluid; WBC, white blood count.

## Data Availability

The raw data supporting the conclusions of this article will be made available by the authors on request.

## Abbreviations

The following abbreviations are used in this manuscript:

AAP	American Academy of Pediatrics
BW	Birth Weight
CRP	C-Reactive Protein
CSF	Cerebrospinal Fluid
EONM	Early-Onset Neonatal Meningitis
EOS	Early-Onset Sepsis
GBS	Group B Streptococcus
GA	Gestational Age
HMC	Hamad Medical Corporation
IRB	Institutional Review Board
LP	Lumbar Puncture
MRC	Medical Research Center
MRI	Magnetic Resonance Imaging
NICU	Neonatal Intensive Care Unit
NICE	National Institute for Health and Care Excellence
RBC	Red Blood Cell
OR	Odds Ratio
WBC	White Blood Cell
WWRC	Women’s Wellness and Research Center
